# The green shield: *Trichoderma's* role in sustainable agriculture against soil-borne fungal threats

**DOI:** 10.1016/j.crmicr.2024.100313

**Published:** 2024-11-14

**Authors:** Mansoor Ahmad Malik, Nusrat Ahmad, Mohd Yaqub Bhat

**Affiliations:** Section of Mycology and Plant Pathology Laboratory, Department of Botany, University of Kashmir, Srinagar 190006, India

**Keywords:** Biocontrol, *Trichoderma* species, Inhibition percentage, Soil borne pathogens and dual culture technique

## Abstract

Soil-borne pathogenic fungi are a major agricultural concern, leading to significant decreases in plant yield. Chemically controlling these plants imposes environmental threats that could potentially endanger both humans and other animals. Therefore, employing biological methods in plant disease control represents a more effective alternative approach. The objective of this study was to isolate *Trichoderma* species from soil samples and evaluate their in vitro biocontrol efficacy against fungal pathogens viz. *Fusarium oxysporum, Aspergillus niger, Rhizoctonia solani, Cladosporium cladosporioides, Alternaria alternata, Penicillium citrinum, Curvularia lunata, Fusarium metavorans, Aspergillus flavus, Penicillium chrysogenum, Nigrospora sphaerica,* and *Fusarium solani.* The biocontrol testing efficacy of the isolates against various fungal pathogens was assessed using the dual culture technique*.* In this investigation various *Trichoderma* species were isolated from 25 soil samples and were tested against 12 soil borne fungal pathogens. The radial growth inhibition of *Trichoderma harzanium* and *Trichoderma viride* varied between (20.18% to 58.13% t), (07.01% to 67.16%) respectively. Furthermore, the culture filtrates of *Trichoderma* species at different concentrations (5%, 10%, 15%, and 20%) caused a significant reduction in the mycelial growth of all the tested fungal pathogens*.* The radial growth inhibition was more by higher concentrations in comparison to low concentrations. In the light of these observations, native *Trichoderma* species seems to be competent biocontrol agents and provide as a sustainable method against disease caused by soil borne plant pathogens.

## Highlights

• Soil-borne pathogenic fungi pose a significant threat to agricultural productivity, resulting in substantial yield losses.
• Traditional chemical methods for controlling these pathogens carry environmental risks, necessitating the exploration of biological alternatives.
• The study aimed to isolate *Trichoderma* species from soil samples and assess their effectiveness in controlling various fungal pathogens.

## Graphical Abstract

**Figure:**
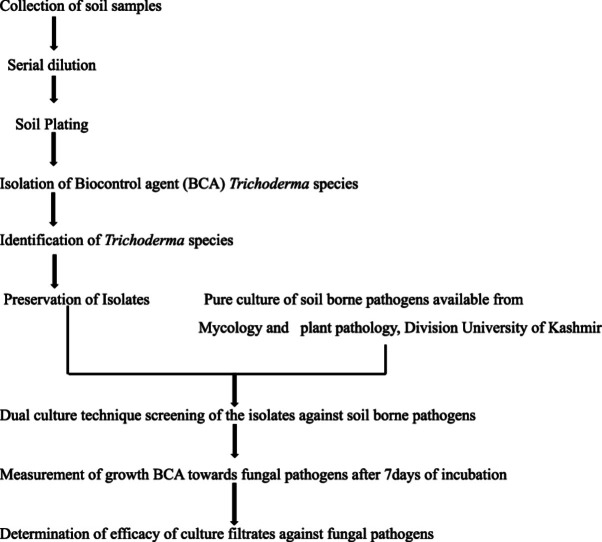

## 1 Introduction

Plant pathogenic fungi residing in the soil contribute to significant crop losses globally (Doehlemann et al., 2017). Agriculture has long grappled with the detrimental impact of various pests and pathogens, resulting not only in diminished crop yields but also in the loss of aesthetic appeal (Khan, 2021). Chemical methods employed to control these pathogens not only disrupt the environment but also undermine ecological balance, diminish soil fertility, and mismanage water resources (Ayala and Rao, 2002; Deshwal et al., 2003; Zhou et al., 2024). Moreover, escalating pesticide expenses, especially in economically disadvantaged regions of worldwide, coupled with consumer preferences for pesticide-free food, have spurred efforts to find substitutes for these products (Bakker et al., 2020). Biological control of plant diseases, particularly those instigated by soil-borne plant pathogens and nematodes, through the utilization of microorganisms, is increasingly perceived as a more natural and environmentally sustainable alternative to conventional chemical treatment methods (Gerhardson, 2002; Ahmad et al., 2021; Pandit et al., 2022). A resurgence of interest in biocontrol among agricultural biologists stems from its environmentally friendly approach to combating weeds, insects, and plant diseases, as well as its long-lasting effects and safety attributes (Anwer, 2017; Haq and Ijaz, 2020; Chaudhary et al., 2024). Interestingly, certain bacterial and fungal antagonists have also demonstrated direct growth-promoting effects when used as crop plant inoculants (Baker and Paulitz, 1996; Deshwal et al., 2003; Orozco-Mosqueda et al., 2021; Elnahal et al., 2022; El-Saadony et al., 2022).

Biological control agents were reported to be key in successfully managing fungal plant pathogens (Ghazanfar et al., 2018). Presently a wide variety of fungi are used as biocontrol agents. Due to its potent antimicrobial properties, *Trichoderma* is the most widely used biocontrol agent against parasitic soil-borne microorganisms (Shahid et al., 2014; Puyam, 2016; Saravanakumar et al., 2021; Yassin et al., 2021; Yassin et al., 2022). Reports state that over 60% of biofungicides are produced by various strains of *Trichoderma* (Sood et al., 2020). *Trichoderma* species have proven to be effective as biocontrol agents against diseases caused by a variety of *Alternaria* species, as well as *Fusarium oxysporum, Rhizoctonia solani, Pythium aphanidermatium, Fusarium culmorum, Gaeumannomyces graminis* var. tritici, *Sclerotium rolfsii, Phytophthora cactorum*, and *Botrytis cinerea* (Kuçuk et al., 2003). *T. viride* and *T. harzianum* may be used as biological control agents to fight against strains of phytopathogenic fungi such as *Fusarium oxysporum, Alternaria alternata*, and *Fusarium solani* (Ramirez-Carino et al., 2020; Yassin et al., 2022).Using the food poisoning technique, Alsubaie et al. (2024) evaluated the antagonistic efficiency of *D. caatingaensis* as a possible biocontrol agent for efficient management of *Fusarium oxysporum, F. solani, F. proliferatum,* and *F. verticillioides*. The most commonly isolated strain was *Fusarium oxysporum*, which was followed by strains of *F. solani, F. proliferatum*, and *F. verticillioides*. The strain of *F. solani* exhibited the least severity of the disease, while the strain of *Fusarium proliferatum* was the most severe.

Through a variety of defence mechanisms, such as mycoparasitism and antibiosis, *Trichoderma* spp. can create a variety of secondary metabolites that provide protection against phytopathogenic fungi and achieved this by either inducing resistance and plant defense reactions (Odebode, et al., 2006; Sood et al., 2020; Yassin et al., 2022). *Trichoderma* species not only generate potent antibiotics but also synthesize mycotoxins and over 100 metabolites possessing antibiotic properties (Sivasithamparam et al., 2002; Sharma et al., 2019; Sood et al., 2020; Hamrouni et al., 2021). Antibiotics like herzianolide, trichodermin, and trichodermol are secreted by *Trichoderma* species to produce their antagonistic effects (Ghorbanpour et al., 2018; Kubheka and Ziena, 2022;Yassin et al., 2022). Antibiotics that suppress plant pathogens include gliotoxin, which is produced by *Trichoderma virens* against *Rhizoctonia solani*, which is the cause of plant root rot, and 2,4 diacetylphloroglucinol, which is produced by *Pseudomonas fluorescens* F113 against *Pythium* species, which causes damping off disease (Pal et al., 2006; Prajapati et al., 2020; Walia et al., 2021).Their antibacterial and antifungal properties are attributed to a number of volatile chemicals, including ethylene, acetaldehyde, and acetone (Piechulla et al., 2017; Ghorbanpour et al., 2018; Yassin et al., 2022). Additionally, the enzymes produced by *Trichoderma* species have the potential to hydrolyse proteins, cellulose, hemicellulose, and chitin, which directly suppresses plant diseases (Sharma et al., 2019). Certain *Trichoderma* species produce enzymes with specificity, which allows them to efficiently attack particular plant diseases (Bastakoti et al., 2017). Using GS-MS, Yasin et al. (2022) and Yasin et al. (2021) identified distinct active components with antiphytopathogenic action in *T. viride* and *T. harzianum*. 6-pentyl-α-pyrone, hexadecanoic acid, acetic acid, 2-phenylethyl alcohol, 2-butoxyethyl acetate, 1-methoy-2-propanone, 1-hexadecanol, isopentyl acetate, dioctyl ester, 9-eicosane, cyclooctanol propyl-benzene, cadinene, epizonaren, d-limonene, α-bisabolol, β-farnesene, and propanoic acid, palmitic acid, proyl benzene, oleic acid, caryophyllene oxide, β-eudesmol, 1-pentanol, cholic acid, octadecenoic acid, ethyl benzene, chavicol, diisooctyl phthalate, harzianic acid, dihydroxyacetone, xylene, 2H-pyran-2-one, and 9-eicosane were isolated from different extracts of *T. viride* and *T. harzianum*. According to Vinale et al. (2009), harzianic acid shown efficacy as an anti-mycotic agent against strains of *Rhizoctonia solani, Sclerotria sclerotiorum*, and *Pythium irregulare*. Antimicrobial effectiveness against several fungal strains has also been observed for 2-phenylethyl alcohol, dihydroxyacetone, hexadecanoic acid, and 9-eicosane compounds (Yasin et al., 2021). Numerous bioactive components, including acetic acid, harzianic acid, 6-pentyl-alpha-pyrone and 2-H-pyran-2-one, 2-phenylethyl alcohol, dihydroxyacetone, hexadecanoic acid, and 9-eicosane, may contribute to the anti-fusarial potency of *T. harzianum* acetonic extract (Yasin et al., 2021). According to Zeilinger et al. (2016), alkyl pyrones with antifungal qualities include 2-H-pyran-2-1 and 6-pentyl-alpha-pyrone. Further, 6-pentyl-alpha-pyrone inhibited filamentous phytopathogenic fungus strains' mycelial development, as demonstrated by Ismaiel and Ali (2017). Palmitic acid was the primary active component of the n-hexane extract of *T. viride*, according to Ali and El-Ghonemy (2014).The mycelial growth of many pathogenic fungal strains was shown to be suppressed by substances such as palmitic acid, octadecenoic acid, cholic acid, propanoic acid, β-caryophyllene, limonene, and β-eudesmol (Yassin et al., 2021). Other bioactive components that have been shown to have antifungal efficacy are responsible for the antifungal activity rather than the primary component (palmitic acid) alone. However, the synergistic effect of many bioactive components may be the cause of bioactivity (Khan et al., 2020).

Therefore, *Trichoderma* species have been regarded as a feasible alternative approach to controlling plant diseases. *Trichoderma* species serve more than just controlling the proliferation of pathogenic microbes; they also have diverse applications such as enhancing plant defense mechanisms, promoting rhizosphere colonization, and stimulating plant root growth. The occurrence and impact of fungal pathogens have been escalating in recent years due to largely climate change and shifts in cropping systems. Hence, the present investigation was carried out to isolate, identify and evaluate the antagonistic activities of local strain of *Trichoderma* isolates against some phytopathogenic fungi of economic importance under in vitro conditions.

## 2 Materials and methods

The study was conducted in the Mycology and Plant Pathology Laboratory, Department of Botany, University of Kashmir, Srinagar, India.

### 2.1 Collection of soil sample

**Fig. 1:**
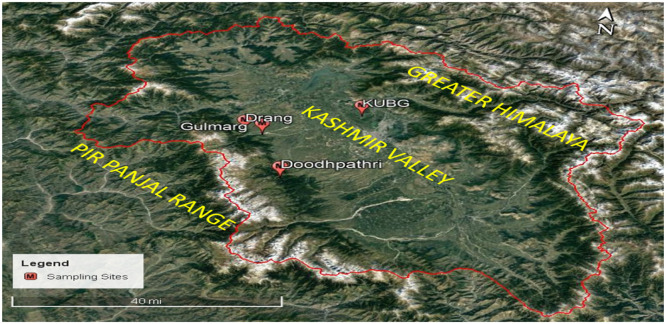
Map showing collection sites.

Twenty-five soil samples were collected from different geographical regions of the Kashmir Himalaya region (Table 1, Fig. 1). Fresh soil samples were collected from rhizospheric zones of medicinal plants like *Swertia petiolata* and *Digitalis purpurea* into clean polythene bags, representing the growth of some *Trichoderma* isolates. Kashmir, having a warm and temperate climate, was also one of the spots for the collection of soil samples. The fungal bioagents, which were locally isolated, were tested for the management of harmful soil fungi under in vitro conditions.

**Table 1:** Latitude and longitude of study sites.

**Study Sites**	**Location**	**Altitude (asl)**	**Latitude**	**Longitude**
**01**	Gulmarg	2650m	34°03ʹ14ʹʹ N	74°23ʹ88ʹʹ E
**02**	Doodhpathri	2850m	33°50ʹ67ʹʹ N	74°35ʹ15ʹʹ E
**03**	Drang	2300m	34°03′32′'N	74°25′57′'E
**04**	Kashmir University Botanical Garden (KUBG)	1591m	34°09ʹ66ʹʹ N	74°50′77′'E

### 2.2 Isolation of biocontrol agent

The collected soil sample from different geographical regions of Kashmir Himalaya was used to isolate *Trichoderma* species. From the serial dilution, soil samples of 1 ml each were poured in selective media Potato Dextrose Agar (PDA) to isolate *Trichoderma* species, and soil plating was performed. The plate was then allowed to incubate for 5-7 days at 25-26°C. Incubation results: fuzzy growth of fungus on the PDA plate was observed, which was also seen after seven days of inoculation.

### 2.3 Identification of *Trichoderma* isolates

After the isolation of all isolates, they were examined under a microscope (Olympus CH20) at the Mycology and Plant Pathology Laboratory, University of Kashmir, Srinagar, for the identification and confirmation of *Trichoderma* species. Microscopic observation of specimens was done by the sticky tape method (Flegel, 1980). Examination of the shape, size, arrangement, and development of conidiophores or phialides or conidia provided a tentative identification of *Trichoderma* species.

### 2.4 Biological control of pathogens using fungal bioagents under *in vitro* conditions

The dual-culture technique was used to evaluate the antagonistic effect of local isolates of the two *Trichoderma* species against soil-pathogenic fungi (Sonnenbichler et al., 1983). For screening different *Trichoderma* species, a 5 mm-diameter disc of active mycelium of the biocontrol fungus was placed 2 cm inside from the edge of the Petri plate. A 5 mm-diameter disc of actively growing pathogenic fungus was also placed at the opposite end of the Petri plate. The samples were then incubated at 25±2°C (Guedez et al., 2009). Plates without the antagonistic fungal disc served as control. All treatments were tested in triplicate. Different parameters such as inhibitory effect, colonization percentage, and biological control index were studied.

The inhibitory effect of the biocontrol agent against the selected pathogenic fungi was calculated as per the following formula (Vincent, 1947; Hajieghrari et al., 2008).

$$I=\left[1-\frac{T}{C}\right]\;\times \;100$$

Where C represents the pathogen's radial growth in control and T represents the radial growth of pathogen in the presence of *Trichoderma* isolates.

### 2.5 Colonization percentage

Colonization percentage was calculated as per following formula (Rollen et al., 1999).

$$C=\frac{\mathrm{DTP}}{\mathrm{DE}}\times \;100$$

Where C = Colonization percentage of *Trichoderma* species over different fungal pathogens, DTP is the distance travelled by the *Trichoderma* sp. colony on the pathogen colony and DE is the distance between two agar plugs (4cm).

### 2.6 Biocontrol index

The biocontrol index (BCI) depicts the percentage inhibition in the growth of pathogenic fungi which was calculated as per the following formula (Szekeres et al., 2006).

$$\text{BCI}=\frac{A}{B}\;\times \;100$$

Where A is the area of the colony of biocontrol fungus and B is the total area of colony of biocontrol fungus and pathogenic fungus.

### 2.7 Effect of culture filtrates of different *Trichoderma* isolates on the mycelial growth of fungi

The two species of *Trichoderma* were inoculated and cultured in 500-ml conical flasks containing 300 ml of Richard's solution for 20–30 days at 25±2°C in an incubator (Dennis and Webster, 1971). To get cell-free culture filtrates, the filtrates of *Trichoderma* isolates were filtered through Whatman filter paper No. 1, followed by centrifugation at 11000-12,000 rpm for 15-20 minutes (El-Boghadady, 1993). Using the Poisoned Food Technique, the efficacy of culture filtrate of the two *Trichoderma* species against various fungal pathogens was evaluated (Nene and Thapliyal, 1979). Final concentrations of 5%, 10%, 15%, and 20% were obtained by adding 1 ml, 2 ml, 3 ml, and 4 ml of the culture filtrate of each species of *Trichoderma* to the molten PDA media, nearly 20 ml. After solidification of the medium in sterilized Petri plates, 5 mm mycelial discs of pathogenic fungus were inoculated in the center of the sterilized Petri plates. Petri plates in which fungal discs were inoculated on a PDA medium without culture filtrate served as a control. The whole process was carried out three times. The Petri plates were then incubated for 4–8 days at 25°C in an incubator. Percent growth inhibition was calculated as follows (Asalmol et al., 1990).

Percent Growth Inhibition (I) = [C - T/C] x 100

Where “C” denotes the colony diameter (mm) of a pathogen in the control group and “T” stands for colony diameter (mm) in the treatment group.

## 3 Statistical analysis

The statistical analysis of data was done using SPSS 23 (SPSS Inc., Chicago, IL, USA) software. Standard deviation was calculated. ANOVA was used to test the difference between different treatments. Duncan's multiple comparison tests was used to compare all the treatments and differences between individual means at (P≤ 0.05).

## 4 Results

### 4.1 Isolation of biocontrol agent

**Fig. 2:**
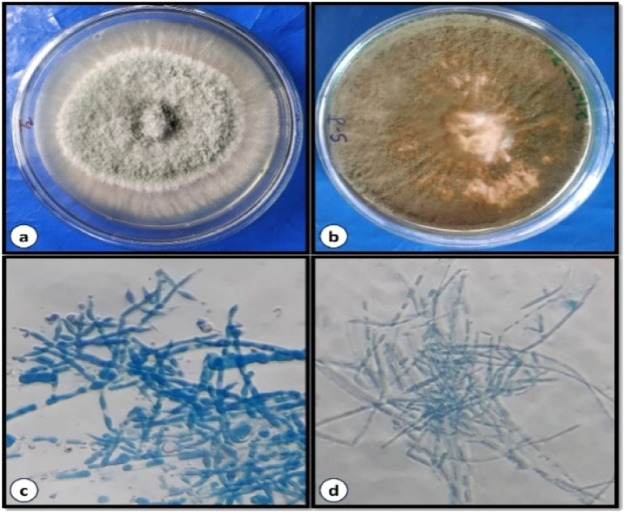
Fungal culture of (a) *Trichoderma viride* and (b) *Trichoderma harzianum* on PDA medium, Microscopic view of *Trichoderma* isolate with repeatedly branched Conidiophore, phialides and rough conidia (400X).

The growth of *Trichoderma* species in PDA plates was observed after 7 days of inoculation. Isolated *Trichoderma* species from soil samples used as biocontrol agents are represented in Fig. 2 (a–d). Out of 25 soil samples tested, the *Trichoderma* species were isolated in 10 soil samples. The green or white-coloured fuzzy form growth indicated the *Trichoderma* species and was confirmed by the microscopic examination (Fig. 2 a–d).

### 4.2 The efficacy of two *Trichoderma* species against pathogenic fungi using dual culture experiments

The efficacy of two species of *Trichoderma, Trichoderma harzianum* and *Trichoderma viride* were assessed using dual culture experiments against some pathogenic soil fungi, viz. *Fusarium oxysporum, Aspergillus niger, Rhizoctonia solani, Cladosporium cladosporioides, Alternaria alternata, Penicillium citrinum, Curvularia lunata, Fusarium metavorans, Aspergillus flavus, Penicillium chrysogenum, Nigrospora sphaerica* and *Fusarium solani* under *In vitro* conditions (Table 2). The effect of these *Trichoderma* species was observed on radial growth inhibition, colonization percentage and biocontrol index. This study revealed that both species of *Trichoderma* significantly inhibited every fungal pathogen that was examined.

**Table 2:** Efficacy of *Trichoderma* species on mycelium growth inhibition, colonization percentage and Biocontrol index (BCI) of some pathogenic soil fungi.

Treatment	Mycelial growth inhibition (%)		Colonization percentage (%)		Biocontrol index (%)	
Fungal isolate	*T. harzianum*	*T. viride*	*T. harzianum*	*T. viride*	*T. harzianum*	*T. viride*
*Fusarium oxysporum*	49.54±1.52^b^	56.84± 2.51^a^	21.33±3.05^a^	29.33±8.08^ab^	65.22±1.33^b^	55.30±4.88^a^
*Aspergillus niger*	41.74±2.08^b^	39.51±1.15^a^	42.00±2.00^a^	45.66±1.15^b^	71.45±1.97^a^	64.44±4.99^b^
*Rhizoctonia solani*	55.40±1.52^a^	48.13±2.51^a^	67.33±3.05^a^	70.66±1.15^a^	54.72±4.01^a^	50.64±0.52^b^
*Cladosporium cladosporioides*	20.18±2.08^a^	07.01±1.15^b^	23.33±1.15^a^	26.00±2.00^a^	65.31±0.97^a^	69.42±0.70^b^
*Alternaria alternata*	23.33±4.16^a^	57.75±1.15^a^	28.00±2.00^a^	32.00±2.00^ab^	67.40±1.15^a^	72.61±6.65^a^
*Penicillium citrinum*	21.29±1.00^b^	53.77±2.08^a^	35.33±1.15^a^	55.33±3.05^b^	51.39±2.13^a^	65.22±1.33^b^
*Curvularia lunata*	58.13±2.00^a^	56.42±0.57^a^	45.33±1.15^a^	47.33±.3.05^ab^	52.71±0.60^a^	51.47±1.08^a^
*Fusarium metavorans*	57.33±1.00^a^	57.67±1.15^a^	44.00±2.00^a^	46.66±2.30^a^	66.07±1.48^a^	65.11±0.90^a^
*Aspergillus flavus*	44.14±1.52^b^	67.16±1.00^a^	62.00±2.00^a^	60.66±1.15^a^	77.24±1.82^c^	70.90±2.27^b^
*Penicillium chrysogenum*	50.00±1.52^a^	41.51±1.00^b^	64.66±1.15^a^	44.33±6.42^b^	66.91±1.57^a^	62.60±1.16^a^
*Nigrospora sphaerica*	44.58±1.00^b^	59.87±1.00^a^	71.33±1.15 ^a^	56.00±2.00^b^	65.22±1.33^b^	55.30±4.88^a^
*Fusarium solani*	48.96±1.00 ^b^	52.96±1.00^a^	65.00±1.00 ^a^	24.33±1.15^b^	71.45±1.97^a^	64.44±4.99^b^

Data shown in Mean ± SD, mean with the same superscript in the same column are not significantly different while those with different superscripts are significantly different using the Duncan's multiple comparison test at (p ≤ 0.05).

#### 4.2.1 Effect of *Trichoderma* species on radial growth inhibition of fungal pathogens

**Fig 3:**
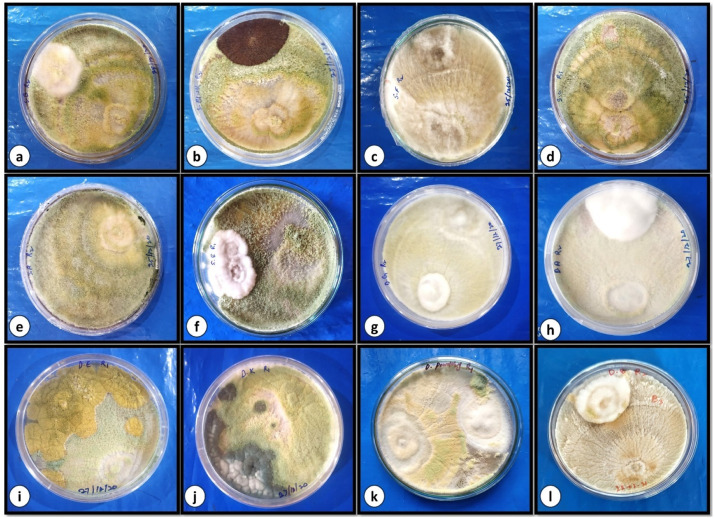
Dual culture of *Trichoderma harzanium* against, (a) *Fusarium oxysporum,* (b) *Aspergillus niger,* (c) *Rhizoctonia solani,* (d) *Cladosporium cladosporioides,* (e) *Alternaria alternata,* (f) *Penicillium citrinum,* (g) *Curvularia lunata,* (h) *Fusarium metavorans,* (i) *Aspergillus flavus,* (j) *Penicillium chrysogenum,* (k) *Nigrospora sphaerica,* and (l) *Fusarium solani.*

**Fig 4:**
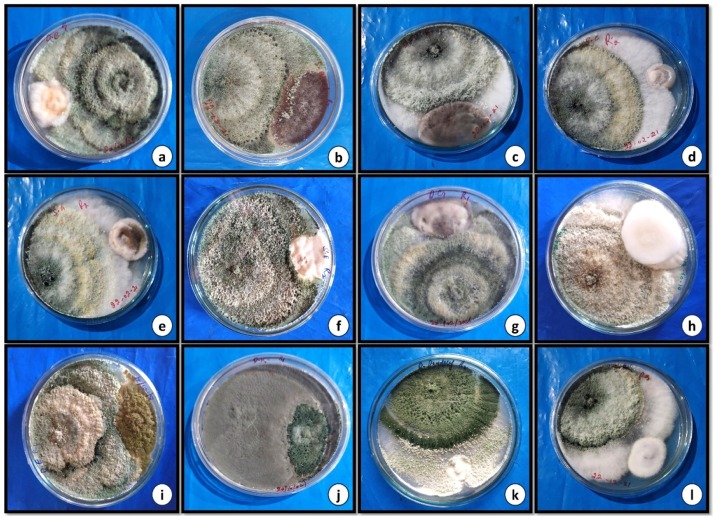
Dual culture of *Trichoderma viride* against, (a) *Fusarium oxysporum,* (b) *Aspergillus niger,* (c) *Rhizoctonia solani,* (d) *Cladosporium cladosporioides,* (e) *Alternaria alternata,* (f) *Penicillium citrinum,* (g) *Curvularia lunata,* (h) *Fusarium metavorans,* (i) *Aspergillus flavus,* (j) *Penicillium chrysogenum,* (k) *Nigrospora sphaerica,* and (l) *Fusarium solani.*

According to the study, every isolate of *Trichoderma* species significantly inhibited the radial development of the pathogenic soil fungi that were put to the test. *Trichoderma viride*, one of the two species studied, was shown to be more effective than *Trichoderma harzianum* at preventing the radial development of the tested fungal pathogens. The radial growth inhibition of *Trichoderma harzanium* varied between 20.18% to 58.13% respectively for different fungal pathogens with highest inhibition of *Curvularia lunata* (58.13%) followed by inhibition in radial growth of *Fusarium metavorans* (57.33%), *Rhizoctonia solani* (55.40%), *Penicillium chrysogenum* (50.00%), *Fusarium oxysporum* (49.54%), *Fusarium solani* (48.96%), *Nigrospora sphaerica* (44.58%), *Aspergillus flavus* (44.14%), *Aspergillus niger* (41.74%), *Alternaria alternata* (23.33%), *Penicillium citrinum* (21.29%) respectively and least effective against Cladosporium cladosporioides (20.18%). Similarly, the radial growth inhibition of *Trichoderma viride* varied between 07.01%. to 67.16% The highest reduced growth inhibition was in *Aspergillus flavus* (67.16%) followed by *Nigrospora sphaerica* (59.87%), *Alternaria alternata* (57.75%), *Fusarium metavorans* (57.67%), *Fusarium oxysporum* (56.84%), *Curvularia lunata* (56.42%), *Penicillium citrinum* (53.77%), *Fusarium solani* (52.96%), *Rhizoctonia solani* (48.13%), *Penicillium chrysogenum* (41.51%), *Aspergillus niger* (39.51%) and the least effective (07.01%) against *Cladosporium cladosporioides* (Table 2, Figs. 3 a-l and 4 a-l).

#### 4.2.2 Colonization percentage of *Trichoderma* species against some pathogenic soil fungi

In the current research, impact of biocontrol fungi *Trichoderma harzanium* and *Trichoderma viride* were tested for their efficacy on the colonization percentage of pathogenic soil fungi after 7 days of inoculation (Table 2, Fig. 3 a-l and 4 a-l). The study demonstrated that all *Trichoderma* species had considerably different colonization rates against various fungal infections. *Trichoderma harzianum* was having highest colonization percentage against *Nigrospora sphaerica* (71.33%). However, *Trichoderma viride* revealed highest colonization percentage against *Rhizoctonia solani* (70.66%), respectively. The fungus *Trichoderma harzianum* depicted different colonization percentage in the range of 21.33% to 71.33%. The highest percentage colonization against *Nigrospora sphaerica* was 71.33% followed by *Rhizoctonia solani* (67.33), *Fusarium solani* (65.00%), *Penicillium chrysogenum* (64.66%), *Aspergillus flavus* (62.00%), *Curvularia lunata* (45.33%), *Fusarium metavorans* (44.00%), *Aspergillus niger* (42.00%), *Penicillium citrinum* (35.33%), *Alternaria alternata* (28.00%), and *Cladosporium cladosporioides* (23.33%), respectively with minimum colonization percentage against *Fusarium oxysporum* (21.33%). The colonization percentage of *Trichoderma viride* varied between 24.33% to 70.66% respectively for different fungal pathogens with highest colonization percentage observed against *Rhizoctonia solani* (70.66%) followed by *Aspergillus flavus* (60.66%), *Nigrospora sphaerica* (56.00%), *Penicillium citrinum* (55.33%), *Curvularia lunata* (47.33%), *Fusarium metavorans* (46.66%), *Aspergillus niger* (45.66%), *Penicillium chrysogenum* (44.33%), *Alternaria alternata* (32.00%), *Fusarium oxysporum* (29.33%) and *Cladosporium cladosporioides* (26.00%) respectively and least colonization percentage by *Trichoderma viride* was shown against *Fusarium solani* (24.33%).

#### 4.2.3 Biocontrol index (BCI) of *Trichoderma* species against some pathogenic soil fungi

The biocontrol index measures the percentage inhibition of the growth of pathogenic fungi by fungal biocontrol agents. The higher the biocontrol index, the more efficient the biocontrol agent is against the pathogenic fungi. The results (Table 2, Figs. 3 a-l and 4 a-l) showed that *T. harzanium* showed the highest biocontrol index after five days of incubation, followed by *T. viride*. The biocontrol index of *T. harzanium* against different fungal pathogens varied between 51.39% to 77.24% with highest biocontrol index against *Aspergillus flavus* (77.24%) followed by *Aspergillus niger* (71.45%), *Fusarium solani* (71.45%), *Alternaria alternata* (67.40%), *Penicillium chrysogenum* (66.91%), *Fusarium metavorans* (66.07%) *Cladosporium cladosporioides* (65.31%), *Nigrospora sphaerica* (65.22%), *Fusarium oxysporum* (65.22%), *Rhizoctonia solani* (54.72%), *Curvularia lunata* (52.71%) and *Penicillium citrinum* (51.39%) respectively*.* Similarly, the biocontrol index of *T. viride* against different fungal pathogens varied between 50.64% to 72.61% with highest biocontrol index against *Alternaria alternata* (72.16%) followed by biocontrol index against *Aspergillus flavus* (70.90%), *Cladosporium cladosporioides* (69.42%), *Penicillium citrinum* (65.22%), *Fusarium metavorans* (65.11%), *Fusarium solani* (64.44%), *Aspergillus niger* (64.44%), *Penicillium chrysogenum* (62.60%), *Nigrospora sphaerica* (55.30%), *Fusarium oxysporum* (55.30%), *Curvularia lunata* (51.47%) and least biocontrol index was observed against *Rhizoctonia solani* (50.64%).

### 4.3 Effect of culture filtrates of *Trichoderma* species against soil pathogenic fungi

**Fig. 5:**
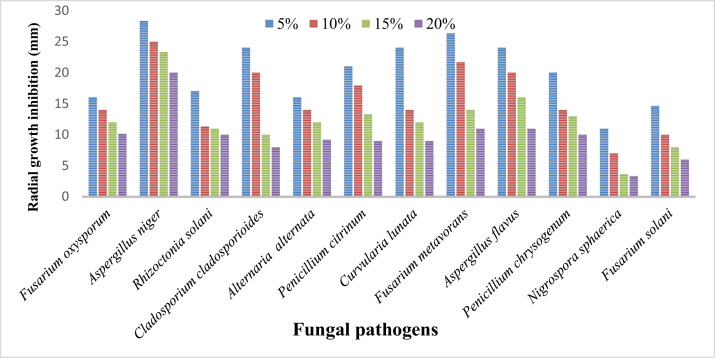
Effect of various concentrations of culture filtrates of *Trichoderma harzianum* on the mycelial growth inhibition of some pathogenic fungi.

**Fig. 6:**
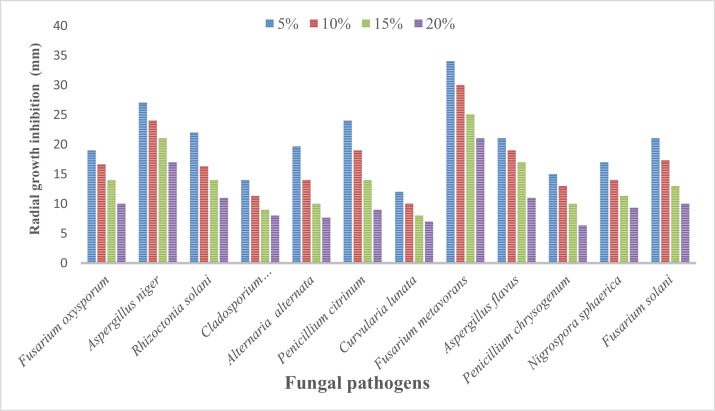
Efficacy of various concentration culture filtrate of *Trichoderma viride* on the mycelial growth inhibition of some soil pathogenic fungi.

At various concentrations (5%, 10%, 15%, and 20%) the culture filtrate of all *Trichoderma* species, viz. *Trichoderma harzanium* and *Trichoderma viride* were tested for inhibition in the radial growth of soil pathogenic fungi, viz. *Fusarium oxysporum, Aspergillus niger, Rhizoctonia solani, Cladosporium cladosporioides, Alternaria alternata, Penicillium citrinum, Curvularia lunata, Fusarium metavorans, Aspergillus flavus, Penicillium chrysogenum, Nigrospora sphaerica* and *Fusarium solani.* The experimental studies revealed strong inhibition of radial development in growth by all concentrations of the culture filtrate of biocontrol fungi *Trichoderma* species. The radial growth inhibition was more by higher concentrations in comparison to low concentrations (Table 3, Table 4; Fig. 5, Fig. 6).

**Table 3:** Effect of various concentrations of culture filtrates of *Trichoderma harzianum* on the mycelial growth inhibition of some pathogenic fungi.

Treatment Fungal isolates	Mycelial growth inhibition (mm)				
	5%	10%	15%	20%	Control
*Fusarium oxysporum*	16.00±1.00^c^	14.00±1.00^b^	12.00±1.00^a^	10.16±0.76^a^	51.66±1.52^d^
*Aspergillus niger*	28.33±1.52^c^	25.00±1.00^b^	23.33±1.52^b^	20.00±1.00^a^	64.33±1.15^d^
*Rhizoctonia solani*	17.00±1.00^b^	11.33±1.57^a^	11.00±1.00^a^	10.00±1.00^a^	42.00±2.00^c^
*Cladosporium cladosporioides*	24.00±1.00^c^	20.00±1.00^b^	10.00±1.00^a^	8.00±1.00^a^	22.00±2.00^bc^
*Alternaria alternata*	16.00±1.00^c^	14.00±1.00^bc^	12.00±1.00^b^	9.20±1.05^a^	52.00±1.73^d^
*Penicillium citrinum*	21.00±1.00^d^	17.90±0.85^c^	13.33±1.57^b^	9.00±1.00^a^	42.66±2.51^e^
*Curvularia lunata*	24.00±1.00^d^	14.00±1.00^c^	12.00±1.00^b^	9.00±1.00^a^	54.66±0.57^c^
*Fusarium metavorans*	26.33±1.15^d^	21.66±1.52^c^	14.00±1.00^b^	11.00±1.00^a^	61.00±1.00^e^
*Aspergillus flavus*	24.00±1.00^d^	20.00±1.00^c^	16.00±1.00^b^	11.00±1.00^a^	91.00±1.00^e^
*Penicillium chrysogenum*	20.00±1.00^c^	14.00±1.00^b^	13.00±1.00^b^	10.00±1.00^a^	91.66±1.52^d^
*Nigrospora sphaerica*	11.00±1.00^c^	7.00±1.00^b^	3.66±1.52^a^	3.33±1.52^a^	42.66±2.51^d^
*Fusarium solani*	14.66±0.57^d^	10.00±1.00^c^	8.00±1.00^b^	6.00±1.00^a^	53.33±1.52^e^

Data shown in Mean ± SD, mean with the same superscript in the same column are not significantly different while those with different superscripts are significantly different using the Duncan's multiple comparison test at (p ≤ 0.05).

**Table 4:** Efficacy of various concentration culture filtrate of *Trichoderma viride* on the mycelial growth inhibition of some soil pathogenic fungi.

Treatment Fungal isolates	Mycelial growth inhibition (mm)				
	5%	10%	15%	20%	Control
*Fusarium oxysporum*	19.00±1.00^d^	16.66±0.57^c^	14.00±1.00^b^	10.00±1.00^a^	34.00±1.00^e^
*Aspergillus niger*	27.00±1.00^d^	24.00±1.00^c^	21.00±1.00^b^	17.00±1.00^a^	42.66±2.51^e^
*Rhizoctonia solani*	22.00±1.00^c^	16.33±0.57^b^	14.00±1.00^b^	11.00±1.00^a^	43.00±2.64^d^
*Cladosporium cladosporioides*	14.00±1.00^c^	11.33±1.57^b^	9.00±1.00^a^	8.00±1.00^a^	31.66±1.52^d^
*Alternaria alternata*	19.66±0.57^c^	14.00±1.00^b^	10.00±1.00^a^	7.66±0.57^a^	42.33±2.51^d^
*Penicillium citrinum*	24.00±1.00^d^	19.00±1.00^c^	14.00±1.00^b^	9.00±1.00^a^	43.66±1.52^e^
*Curvularia lunata*	12.00±1.00^c^	10.00±1.00^b^	8.00±1.00^a^	7.00±1.00^a^	40.66±1.15^d^
*Fusarium metavorans*	34.00±1.00^d^	30.00±1.00^c^	25.00±1.00^b^	21.00±1.00^a^	51.66±1.52^e^
*Aspergillus flavus*	21.00±1.00^c^	19.00±1.00^bc^	17.00±1.00^b^	11.00±1.00^a^	72.66±2.51^d^
*Penicillium chrysogenum*	15.00±1.00^c^	13.00±1.00^c^	10.00±1.00^b^	6.33±1.15^a^	72.33±2.08^d^
*Nigrospora sphaerica*	17.00±1.00^d^	14.00±1.00^c^	11.33±0.57^b^	9.33±0.57^a^	46.66±1.52^e^
*Fusarium solani*	21.00±1.00^d^	17.33±0.57^c^	13.00±1.00^b^	10.00±1.00^a^	27.66±2.08^e^

Data shown in Mean ± SD, mean with the same superscript in the same column are not significantly different while those with different superscripts are significantly different using the Duncan's multiple comparison test at (p ≤ 0.05).

#### 4.3.1 Effect of culture filtrate of *Trichoderma harzanium* on some soil pathogenic fungi

It was found from the results (Table 3, Fig. 5) that various concentrations (5%, 10%, 15%, 20%) of *Trichoderma harzanium* culture filtrate reduced significantly the mycelial growth of all the isolated test fungal pathogens. Mycelial growth was more effectively inhibited by higher concentrations of culture filtrate than by lower concentrations. The *Trichoderma harzanium* culture filtrates were shown to be most effective by preventing the mycelial growth of *Nigrospora sphaerica* (3.33 mm), *Fusarium solani* (6.00 mm), *Cladosporium cladosporioides* (8.00 mm), *Penicillium citrinum* (9.00 mm), *Curvularia lunata* (9.00 mm)*, Alternaria alternata* (9.20 mm), *Rhizoctonia solani* (10.00 mm), *Penicillium chrysogenum* (10.00 mm), *Fusarium oxysporum* (10.16 mm), *Fusarium metavorans* (11.00 mm), *Aspergillus flavus* (11.00 mm), and *Aspergillus niger* (20.00 mm) respectively as compared to control. The inhibition in the radial growth of *Fusarium oxysporum* varied from 10.16 mm to 16.00 mm and for *Aspergillus niger* from 20.00 mm to 28.33 mm and for *Rhizoctonia solani* from 10.00 mm to 17.00 mm respectively. Similarly, the inhibition in radial growth of *Cladosporium cladosporioides* varied from 8.00 mm to 24.00 mm of *Alternaria alternata* varied from 9.20 mm to 16.00 mm of *Penicillium citrinum* varied from 9.00 mm to 21.00 mm. However, the inhibition in radial growth of *Curvularia lunata* and *Fusarium metavorans* varies from 9.00 mm to 24.00 mm and 11.00 mm to 26.33 mm respectively. Similarly, the inhibition in radial growth of *Aspergillus flavus* and *Penicillium chrysogenum* varies from 11.00 mm to 24.00 mm and 10.00 mm. to 20.00 mm. Likewise, the inhibition in radial growth of *Nigrospora sphaerica* and *Fusarium solani* varies from 3.33 mm to 11.00 mm to and 6.00 mm to 14.66 mm respectively.

#### 4.3.2 Efficacy of *Trichoderma viride* culture filtrate on soil pathogenic fungi

It was observed from the results (Table 4, Fig. 6) that culture filtrates of *Trichoderma viride* at different concentrations (5%, 10%, 15%, and 20%) caused a significant reduction in the mycelial growth of all the tested fungal pathogens. Mycelial growth was effectively inhibited by higher concentrations of culture filtrate than by lower concentrations. The *Trichoderma viride* culture filtrates proved to be most effective in preventing the mycelial growth of *Penicillium chrysogenum* (6.33 mm), *Curvularia lunata* (7.00 mm), *Alternaria alternata* (7.66 mm), *Cladosporium cladosporioides* (8.00 mm), *Penicillium citrinum* (9.00mm)*, Nigrospora sphaerica* (9.33 mm), *Fusarium solani* (10.00mm), *Fusarium oxysporum* (10.00 mm), *Rhizoctonia solani* (11.00 mm), *Aspergillus flavus* (11.00 mm), *Aspergillus niger* (17.00 mm), and *Fusarium metavorans* (21.00 mm) as compared to control. The inhibition in radial growth of *Fusarium oxysporum* varied from 10.00 mm to 19.00 mm and for *Aspergillus niger* from 17.00 mm to 27.00 mm and for *Rhizoctonia solani* from 11.00 mm to 22.00 mm respectively by culture filtrates of *Trichoderma viride.* Radial mycelial growth inhibition of *Cladosporium cladosporioides* varied from 8.00 mm to 14.00 mm *Alternaria alternata* varied from 7.66 mm to 19.66 mm *Penicillium citrinum* varied from 9.00 mm to 24.00 mm. However, the inhibition in radial growth of *Curvularia lunata* and *Fusarium metavorans* varies from 7.00 mm to 12.00 mm and 21.00 mm to 34.00 mm respectively. Similarly, the inhibition in radial growth of *Aspergillus flavus* and *Penicillium chrysogenum* varied from 11.00 mm to 21.00 mm and 6.33 mm to 15.00 mm. Likewise, the inhibition in radial growth of *Nigrospora sphaerica* and *Fusarium solani* varied from 9.33 mm to 17.00 mm and 10.00 mm to 21.00 mm to respectively.

## 5 Discussion

The efficacy of *Trichoderma harzianum* and *Trichoderma viride* was assessed using dual culture experiments against 12 pathogenic soil fungi under In vitro conditions. The effect of these *Trichoderma* species was observed with respect to mycelial growth inhibition, colonization percentage and biocontrol index. The results revealed that both the species of *Trichoderma* showed significant inhibitory activity against all the test fungal pathogens. Amongst the two species used *Trichoderma viride* was found more efficient in inhibiting the radial growth of the tested fungal pathogens than *Trichoderma harzianum*.

It was revealed from the study that the colonization percentage of all the *Trichoderma* species against different fungal pathogens varied significantly. The highest colonization percentage was shown by *Trichoderma harzianum* against *Nigrospora sphaerica.* However, least colonization percentage by *Trichoderma harzianum* was shown against *Fusarium oxysporum*. The highest colonization percentage was shown by *Trichoderma viride* against *Rhizoctonia solani* and least against *Fusarium solani*. Similar to our study, Chennappa et al. (2017) assessed the antagonistic capability of *Trichoderma* isolates against various soil-borne diseases, including *Aspergillus, Fusarium, Alternaria*, and *Rhizoctonia solani.* They found that various *Trichoderma* isolates significantly inhibited the tested pathogens*. Trichoderma* (Tri-9) isolates exhibited the highest mycelia growth inhibition against *Sclerotium rolfsii*, and *R. Solani* while as, *Trichoderma* (Tri-2) isolates exhibited the maximum mycelia growth inhibition against *Fusarium.* Likewise, *Trichoderma* (Tri 18) was most hostile to *Aspergillus*. Bunker (2001) reported the impact of volatile and non-volatile compounds produced by *T. harzanium, Gliocladium virens* and *T. aureoviride* against the growth and sclerotia formation of *R. solani*. Sarojini and Nagamani (2011) showed that *T. harzianum* and *T. aeroviride* inhibited the mycelial growth of *R. solani* whereas *T. koningii* and *T. longibrachiatum* were most efficient in reducing the formation of sclerotia of *R. solani.* Similar to our results, Kamaruzzaman et al. (2016) reported the antifungal activities of diverse *Trichoderma* isolates against *Penicillium* sp., *Fusarium oxysporum, Sclerotium rolfsii, Rhizoctonia solani, Aspergillus flavus, Colletotrichum gloeosporiodes* and *Phomopsis vexans* and *Trichoderma* isolates used showed significant variation in mycelial growth by all the *Trichoderma* isolates. However, *T. viride* significantly inhibited the mycelial growth of *C. lunata* compared to the control.

The biocontrol index measures the percentage inhibition of the growth of pathogenic fungi by fungal biocontrol agents. The higher the biocontrol index, the more efficient the biocontrol agent is against the pathogenic fungi. The results showed that *T. harzanium* showed the highest biocontrol index after five days of incubation, followed by *T. viride*. The highest biocontrol index of *T. harzanium* was found against *Aspergillus flavus* and least biocontrol index was observed against *Rhizoctonia solani*. Similar to all results many researchers have observed that *Trichoderma harzianum* is antagonistic to severalof pathogenic fungi (Reetha, et al., 2014; Koka et al., 2017).

The antifungal activity of *Trichoderma* spp. was determined by checking the efficacy of culture filtrates against some selected test soil pathogens. The culture filtrate of all *Trichoderma species,* viz*. Trichoderma harzanium and Trichoderma viride* at various concentrations caused a significant reduction in mycelial growth of all the tested fungal pathogens, viz. *Fusarium oxysporum, Aspergillus niger, Rhizoctonia solani, Cladosporium cladosporioides, Alternaria alternata, Penicillium citrinum, Curvularia lunata, Fusarium metavorans, Aspergillus flavus, Penicillium chrysogenum, Nigrospora sphaerica* and *Fusarium solani.* It was found that mycelial growth was more effectively inhibited by higher concentrations of the biocontrol fungus *Trichoderma harzanium* was shown to be the most efficient followed by *Trichoderma viride* respectively at similar concentrations. The culture filtrates of *Trichoderma* spp. possess specific enzymes or chemicals that are responsible for certain antimycotic activities of these *Trichoderma* species. A significant reduction in mycelial growth by cultures filtrates of *Trichoderma* spp on other pathogenic fungi were also observed by other workers (Calistru et al., 1997; Eziashi*et al*., 2007; Hajieghrari et al., 2008; Mishra, 2010; Thi et al., 2012; Alka and Prajapati, 2017). Kapil and Kapoor (2005) reported that *Trichoderma viride* culture filtrate had an antagonistic effect on *Sclerotinia sclerotiorum*. Similarly, Fajola and Alasoadura (1975) examined the effect of *Trichoderma harzianum* culture filtrates on *P. aphanidermatum* mycelial growth. Likewise, Kakde and Chavan (2011) found that *Curvularia lunata* and *Rhizopus stolonifer* were negatively affected by *Trichoderma viride* and *Trichoderma harzianum*.

## 6 Conclusion

It can be concluded that the isolated *Trichoderma* species reduced the growth of all 12 soil-borne pathogens, viz*. Aspergillus niger, A. flavus, Alternaria alternata, Rhizoctonia solani, Cladosporium cladosporioides, Penicillium citrinum, Curvularia lunata, Fusarium metavorans, F. oxysporum, F. solani, Penicillium chrysogenum, and Nigrospora sphaerica,* significantly at different levels and, therefore, can be incorporated for integrated disease management of soil-borne plant pathogens. Hence, *Trichoderma* species can be used as a potential biocontrol agent against these pathogens. The degree of antagonism varied between and within species of *Trichoderma* against the soil-borne plant pathogens. Therefore, this research can have promising potential in the agricultural field to protect plants affected by various fungal pathogens.

## CRediT authorship contribution statement

**Mansoor Ahmad Malik:** Conceptualization, Methodology, Data curation, Software, Visualization, Writing – original draft. **Nusrat Ahmad:** Data curation, Formal analysis, Writing – original draft, Writing – review & editing. **Mohd Yaqub Bhat:** Writing – review & editing, Conceptualization, Supervision, Investigation.

## Declaration of competing interest

The authors declare that they have no known competing financial interests or personal relationships that could have appeared to influence the work reported in this paper.

## Data Availability

The authors do not have permission to share data.
